# A multi-frequency altimetry snow depth product over Arctic sea ice

**DOI:** 10.1038/s41597-024-04343-4

**Published:** 2025-01-17

**Authors:** Alice Carret, Sara Fleury, Alessandro Di Bella, Jack Landy, Isobel Lawrence, Nathan Kurtz, Antoine Laforge, Jérome Bouffard, Tommaso Parrinello

**Affiliations:** 1 Serco, Frascati, Italy; 2 LEGOS, Université de Toulouse, IRD, CNES, CNRS, UPS, Toulouse, France; 3 ESA-ESRIN, Frascati, Rome, Italy; 4 UiT, Tromsø, Norway; 5 NASA Goddard Space Flight Center, Greenbelt, MD USA

**Keywords:** Physical oceanography, Cryospheric science

## Abstract

Sea ice thickness is an essential variable to understand and forecast the dynamic ice cover and can be estimated by satellite altimetry. Nevertheless, it is affected by uncertainties especially from snow depth, a key parameter to derive it from ice freeboard. We developed a snow depth product based on the differences between CryoSat-2 SAR Ku and IceSat-2 laser altimeters which have different snow penetration capabilities. We compared this product to other existing snow depth products. This comparison indicates correlations all above 0.37 and reaching 0.91 relative to airborne snow radar. We estimate the snow depth uncertainties of our product to be 0.06 m on average. Combining two frequencies to simultaneously measure ice and total freeboard reduces the uncertainties in ice thickness by around a factor of two compared with a single-frequency solution combined with a snow depth climatology. The lack of *in situ* measurements is a limiting factor to assess the advantages of coincident CryoSat-2 and ICESat-2 acquisitions. Validation data are of major importance for upcoming missions.

## Background & Summary

Recent studies predict that the Arctic summer sea ice will disappear before the middle of this century and possibly as early as the 2030s^1^, even earlier than predicted by the sixth assessment report of the IPCC^2^. The absence of sea ice would further increase the Arctic Amplification phenomenon^3^ and its consequences for global climate, the environment and ocean circulation. The continuous reduction in sea ice cover and thickness would also accelerate human activities in the region, whether industrial, transport or tourism-related. Monitoring and tracking the sea ice will therefore become increasingly crucial.

Currently, sea ice monitoring mostly relies on two-dimensional imagery from several sensors (SAR image, optical, thermal, radiometric), as the wide footprint of the images means that complete coverage of the basin can be obtained in a matter of days. While being crucial to monitor important aspects of the sea ice, e.g. its extent and drift, these sensors can not retrieve sea ice thickness (SIT) apart SMOS (Soil Moisture and Ocean Salinity) radiometer which can retrieve thin ice thickness, below 0.5m^4,5^. However, numerous studies have shown that the most relevant parameter for determining the short- and medium-term fate of the sea ice is ice thickness^6–9^. Indeed, the thicker the ice, the more resistant it is to climate warming and short-term weather hazards (storms, heat waves). Knowledge of ice thickness is also crucial for navigation.

The most widely used technique to estimate sea ice thickness is satellite altimetry. The method consists in measuring the ice’s freeboard (FB), i.e. the height of the ice above the local sea surface. Estimating the relative density of ice and water, we can deduce the sea ice thickness. However, the problem is complicated by the fact that sea ice is usually covered by a layer of snow that can reach several tens of centimeters^10,11^. The weight of the snow pushes the ice into the water and alters the hydrostatic balance. Its presence can also modify the freeboard measurements, depending on the snow penetration of the radar or lidar frequency used.

Beyond the altimetry measurement itself, one of the main sources of uncertainty in measuring sea ice thickness lies in the lack of knowledge of snow cover properties like its depth and density which can lead to errors up to 50% of the SIT (see the last section). The other major source of uncertainty, with a similar order of magnitude, concerns the density of the ice itself.

For a long time, the only snow depth (SD) product used by the community was the climatology aggregated by^12^ (hereafter “W99”). More recently there have been different approaches to develop new snow depth products. First alternatives were based on models which coupled ice drift observations with snow precipitation and wind reanalysis data (PIOMAS, NESOSIM, SnowModel-LG, …). Space-borne passive microwave retrievals have also been investigated with AMSR-E and then AMSR-2 instruments^13–15^.

With the launch of the SARAL/AltiKa mission, an approach based on satellite altimetry has been proposed by^16^. This methodology uses dual-frequency altimetry measurements, Ku-band for CryoSat-2 (CS2) and Ka-band for Saral, and exploits the distinct dielectric properties exhibited by the snow at these two frequencies. Ku-band radar signal has been shown to penetrate cold and dry snow in laboratory tests^17^, thus scattering at the snow/ice interface, whereas other studies^16,18^ have suggested that the penetration of Ka-band radar or laser altimetry is expected to be null or limited to a few centimetres. However, there are some specific properties inherent to snow that can limit the penetration of Ku-band radar waves into the snow layer, such as salinity, grain size, moisture and temperature^19^. The radar-scale roughness of the air-snow interface also impacts the transmission of Ku- and Ka-band radar waves into the snowpack^20^. The impact of these snow properties on radar penetration at different frequencies is still under investigation and needs the continuous acquisition of coincident *in situ* data in different snow conditions.

Different approaches to generate Ka-Ku snow depth products have been proposed by^16,21,22^. A major limitation of SARAL data for deriving snow depth is a maximum latitudinal coverage of 81.5° N/S. Solutions based on radar Ku and lidar have been first investigated by^23^ over winter 2018-2019, with the advantage that the ICESat-2 laser altimeter covers the polar regions up to 88° N/S. Later on, the same team used the same methodology to document the snow depth inter-annual variability^24^.

In this context, snow depth is still an important research question. The objective of this study is to present a new snow depth product obtained from CS2 Ku-band and IS2 laser altimetry (the LEGOS product) covering the period 2018–2021. We consider different altimetry processing approaches and cross-compare them with freely available snow products, including all the data from models and satellite measurements mentioned above as well as solutions not yet published but developed as part of the ESA Polar + Snow on Sea Ice project (see Section Source data). Additionally, we investigate the impact of combining Ku-band and laser altimetry for the estimation of sea ice thickness uncertainties. This study focuses on a monthly gridded snow depth product as CryoSat-2 and ICESat-2 are not on the same orbit. However in July 2020 and for 2 years, as part of the CRYO2ICE project, the CS2 orbit was raised by 900 meters to periodically coincide with some IS2 tracks for thousands of kilometers over the Arctic Ocean. These colocalised tracks are an opportunity to study finer temporal and spatial scales.

This new product and alternative solutions are also assessed against all the *in situ* measurements we were able to gather: airborne campaigns, drifting buoys, moorings. Some of these *in situ* data do not include direct measures of snow depth and thus we had to combine them with freeboard or draft measurements to perform meaningful comparisons.

This paper is organized as follows. The next section presents all the data used: altimetry, *in situ* and other snow depth products. The methods section explains how is computed our snow depth product. Data records section presents the dataset produced. The technical validation section assesses this product against all datasets described and provide an analysis of the uncertainties.

## Source Data

This section describes all the datasets used in this paper. It first presents the altimetry data used for our snow depth solution and the other existing dual-frequency altimetry solutions using CS2/IS2 or CS2/Saral combinations. We then present very different solutions, based on radiometers or models, to which we will compare the altimetric solution. These solutions will be assessed using the *in-situ* measurements described next. All these data are summarised in a final table (Table 1).

**Table 1:** Summary of the data used in this study.

	Considered period (Oct-April)	Format	Spatial resolution	Temporal resolution	Measurement Type	Measure
IS2	2018–2022	along-track	24 m		Satellite laser altimetry	Total FB
		gridded	25 km	monthly		
CS2	2018–2022	along-track	300 m	20hz	Satellite radar altimetry	Radar FB
		gridded	25 km	monthly		
OIB	04/2019	along-track	40 m		airborne lidar +snow radar	TotalFB + SD
ICEBird	04/2019	along-track	Every 4-5 m		airborne EM31 + snow radar	TotalFB + SD
BGEP	2018–2021	point	/	daily	moorings	Ice Draft
AMSR	2020–2022	gridded	25 km	daily	radiometry	SD
PIOMAS	2020–2022	gridded	25 km	monthly	model	SD
W99m	climatology	gridded	25 km	monthly	*In situ*	SD
LaKu LEGOS	2018–2021	gridded	25 km	monthly	Satellite altimetry	SD
KaKu LEGOS	2018–2021	gridded	25 km	monthly	satellite alti	SD
LaKu UiT	2018–2021 (Nov - Apr)	gridded	50 km	monthly	satellite alti	SD
KaKu UiT	2018–2021 (Nov - Apr)	gridded	50 km	monthly	satellite alti	SD
LaKu UoL	2018–2021	gridded	25 km	monthly	satellite alti	SD
KaKu UoL	2013–2021	gridded	25 km	monthly	satellite alti	SD
LaKu Kacimi & Kwok, 2022	2018–2021	gridded	25 km	monthly	satellite alti	SD

### Altimetry data for presented solution

#### CryoSat-2

CryoSat-2 (CS2) was launched in April 2010^25^ and it is still in operation as of 2024. Its main objective was to monitor polar regions and to study the cryosphere. With an inclination of about 92 degrees, it covers latitudes up to 88° N/S. It carries SIRAL (SAR Interferometry Radar Altimeter), the first synthetic interferometric altimeter in space which has enabled major progresses in sea ice thickness estimation. This instrument operates in Ku-band with 3 acquisition modes: the Low Resolution Mode (LRM) which consists of conventional pulse-width limited altimetry with a footprint of ~7 km radius,the Synthetic Aperture Radar (SAR) which enables to reduce the footprint size along the track to about 300 m and the SAR interferometric (SARIN) mode, a SAR mode where the echoe is received by 2 antennas allowing to solve the left/right ambiguities and determine the across-track location of the main backscatter.

The SAR Altimetry mode is the most widely used mode over sea ice, but SARIN is also used over the entire Western Arctic coastline (from Alaska to Svalbard), parts of the Norwegian and Russian coastlines, dedicated boxes in the Arctic Ocean, and all along the Antarctic continental margins (for more details about the CS2 acquisition mode mask and its evolution throughout the mission lifetime, see https://earth.esa.int/eogateway/instruments/siral/geographical-mode-mask).

In this study we used the L2 product which we had calculated by the ESA GPOD (Grid Processing On Demand) system using the SAMOSA+ physical retracker (hereafter called SAM+). We have then processed it into a sea ice product using the LEGOS sea ice processor^26,27^. Finally, another LEGOS product - hereafter called TFMRA50 (Threshold First Maximum Retracker Algorithm) - is used. It is obtained by applying the empirical retracker TFMRA^28^ with a retracking point selected at the 50% power threshold on the waveform leading edge. The other treatment steps (lead/floes classification, corrections, SSHA interpolation, etc) are described in^27^.

In this paper only snow depth results using the SAMOSA+ retracker are shown because they provide better results than for TFMRA50 (see section about the technical validation).

#### ICESat-2

ICESat-2 (Ice, Cloud, and land Elevation Satellite 2 - IS2), developed by NASA, was launched on September 15, 2018 into a near-circular, near-polar orbit with an altitude of approximately 496 km. It was designed to measure ice sheet elevation and sea ice thickness, as well as land topography, vegetation characteristics, and clouds^29^. It was expected to operate for three years but the mission operation has recently been extended, with current estimates of fuel potentially lasting until the mid 2030’s.

The main instrument on board IS2 is the Advanced Topographic Laser Altimeter System (ATLAS), a space-based lidar. ATLAS measures the travel time of laser photons from the satellite to Earth and back^30^.

ATLAS emits visible laser pulses at 532 nm wavelength. It generates six beams arranged in three pairs in order to better determine the surface’s slope and provide more ground coverage. Each beam pair is 3.3 km apart across the beam ground track, and each beam in a pair is separated by 2.5 km along the beam track. In a pair the beams transmit different energies: the strong beams have about 4 times the weak beams energies and are thus adapted to the ice backscatter variability. A beam pair track is separated by about 90 m. In this study we used only the middle strong beam which is the most centred on CS2 track.

The elevation measurements are taken about every 0.70 m along the satellite’s ground path with a laser footprint of about 11 m of diameter. IS2 measures the total freeboard, which corresponds to the height of the ice freeboard plus the snow depth.

In this paper we use the ATL10 product version 6^31^ downloaded from NSIDC (https://nsidc.org/data/).

### Other snow depth products from altimetry data

#### KaKu LEGOS

The Altimetry Snow Depth (ASD or KaKu LEGOS) product was developed at the LEGOS laboratory^16,22^. The principle is based on the differences between the CS2 Ku-band measurements - which mostly penetrates the snow layer - and the SARAL Ka-band measurements - which hardly penetrates the snow layer. In order to minimise the impact of the surface roughness, they try to make the footprint of SARAL LRM and CS2 SAR and SARIN acquisitions comparable by replacing CS2 SAR/SARIN data with pseudo-LRM measurements provided in the official ESA Baseline-C Geophysical Ocean Product^32^ (GOP).

The KaKu LEGOS product is available in both hemispheres over the period 2013 to 2023. We used monthly gridded data with a spatial resolution of 25 km.

The KaKu LEGOS product has been validated in the Arctic against various snow products^22^ including *in situ* data with quite good agreement (correlation of 0.66 with OIB).

### KaKu and LaKu from University of Tromsø (KaKu and LaKu UiT)

As part of the ESA Polar + Snow on Sea Ice project (Polar + SSI), the University of Tromsø, Norway, has developed two independent snow depth products from dual-altimeter combinations. Both products use Baseline-D CS2 SAR and SARIn level 1B observations combined with either Saral-AltiKa GDR provided by AVISO or with the IS2 ATL20 gridded sea ice freeboard product version 3.

Here again, a key assumption made to compute the snow depth is that the radar freeboard from CS2 Ku-band represents the height of the snow/ice interface above sea level, whereas the radar freeboard from SARAL/AltiKa or from IS2 represents the height of the air/snow interface.

The methodology for obtaining radar freeboards from CS2 and Saral/AltiKa waveforms is based on LARM, a physical retracker presented in^20,33^. This retracker offers the possibility to handle the small-scale roughness parameter which greatly varies for acquisitions over ice floes and leads. It also has the distinctive feature of proposing a lognormal surface backscatter model that is more representative of floe backscatter distribution than the Gaussian model widely used in physically-based retrackers.

The along-track radar freeboards are gridded to a spatial resolution of 50 km, on a monthly basis, and the IS2 laser freeboards are resampled to the same grid, using “drop in a bucket” averaging.

Finally, the UiT snow depth products are generated from the difference between gridded Saral or IS2 and CS2 radar freeboards, accounting for the delayed Ku-band wave propagation in snow following^21^. Snow depths are available for the winter months of 2018–2021.

### KaKu and LaKu from University of Leeds (KaKu and LaKu UoL)

As part of the ESA Polar + Snow on Sea ice project (Polar + SSI), CPOM at the University of Leeds developed two independent snow depth products from multi-satellite synergies. The KaKu solution is based on the Dual-altimetry Snow Thickness (DuST) solution presented in^21^. In order to overcome the mode differences between Saral LRM and CS2 SAR this methodology utilises airborne data from NASA Operation IceBridge to calibrate the satellite freeboards, thereby empirically correcting for the combined effects of surface roughness, difference in satellite footprints, and snow penetration. Snow depth is then estimated by differencing calibrated Ka and Ku gridded freeboards. Within Polar + SSI, the original DuST methodology was extended to include available Sentinel-3A/B data^34^ in the Ku-band satellite dataset. Also, the calibration with IceBridge data has been extended to 2019. Snow depths are available as monthly-gridded products for the winter months (October to April) 2013–2022.

The DuST methodology was also applied to data from CS2/S3A&B and IS2 in the context of the Polar + SSI project. Here, calibrated radar freeboards were subtracted from ATL20 IS2 monthly gridded sea ice freeboards version 1 to estimate snow depth. This dataset, covering two winters (November-April) 2018–2020, has not yet been published nor validated.

### Kacimi and Kwok, 2022 product

^24^Developed a product according to a freeboard differencing methodology described in^23^. They based their approach on CS2 Baseline D L1 from the ESA portal and IS2 ATL10 version 4. They compute CS2 freeboards using a centroid retracker with an adaptive threshold according to the backscatter^35^. To compute freeboard differences they consider a maximum delay of 10 days and a spatial distance of 75 km. Data cover three Arctic winters (October to April) from 2018 to 2021 and are available at https://icesat-2.gsfc.nasa.gov/sea-ice-data/kacimi-kwok-2022.

### Non altimetry snow depth products

#### Bremen AMSR-2 (AMSR)

The Advanced Microwave Scanning Radiometer 2 (AMSR-2) is part of the SHIZUKU satellite from the Japan Aerospace Exploration Agency, which was launched in May 2012. Several studies have shown that snow depth on sea ice can be estimated from AMSR-E (the predecessor of AMSR-2) and AMSR-2, with limitations depending on the season or type of sea ice. This is achieved by comparing brightness temperatures at different frequencies, which vary for ice and snow.

We use the snow depth gridded product from the Bremen Institute^14^, https://doi.pangaea.de/10.1594/PANGAEA.902747) with a stereo-polar grid of about 25 km that we re-projected on the EASE2 format. It covers the winter from November to April for first-year ice and from March to April for multi-year ice.

#### PIOMAS

The Pan-Arctic Ice Ocean Modeling and Assimilation System (PIOMAS) is developed at the Polar Science Center (PSC). It provides Arctic sea ice thickness and concentration, snow depth, ice growth rate, ocean surface salinity from 1978 to present at a monthly temporal resolution, from 45°N to 90°N, and spatial resolution of about 25 km. Data are available at https://pscfiles.apl.washington.edu/zhang/PIOMAS/data/v2.1/other/.

PIOMAS assimilates satellite ice concentration data and observations of sea surface temperature. It is a thickness and enthalpy distribution model. The snow depth is obtained using a snow-thickness distribution equation after solving sea-ice momentum equations, heat equation and ice-thickness distribution equation^36^. For further information the method is explained in^37,38^ present in detail the model and its uncertainty.

#### Warren 99 modified climatology (W99m)

The Warren 99 climatology is the most widely used product for snow depth^12^. It was built using measurements from 1954 to 1991 taken once to three times a month at 31 soviet drifting stations over multi-year ice (MYI). It results in monthly iso-snow depth curves that we have converted to a monthly gridded climatology with a 25 km resolution.

As a result of climate change, snow depths have decreased considerably since then, particularly on the new-year ice and this climatology became outdated.A reduction by a factor 2 of the snow over first year ice (FYI) was hypothesized by^39^ from one year of airborne survey and supported by^11^ who looked at inter-decadal changes. Thus, for the recent period many SIT product developers use a modified version of Warren, which simply consists in dividing by two the snow depth over FYI^40^ first applied this correction. This modified version of Warren, called W99m, has been, and is, widely used to convert altimetry-based radar freeboard measurements to sea ice and thickness estimates (at least for CS2 period).

To calculate this modified version we use the ice type product OSI-403-d from OSISAF (https://osi-saf.eumetsat.int/products/osi-403-d) to distinguish between FYI and MYI.

#### NESOSIM

NESOSIM (NASA Eulerian Snow On Sea Ice Model) is a model which provides the snow depth, the snow volume, the snow density, the snowfall and ice concentration data over the Arctic Ocean^41^. It is forced by snow fall from ERA-I and three other reanalyses, near surface winds from ERA-I, sea ice concentration and sea ice drift. It covers the winter periods since 1992 and it is provided on a 100-km resolution polar stereographic grid. We use the monthly version 1.1 available at https://zenodo.org/records/13754787 that we converted to a monthly gridded dataset on a 25-km grid.

#### SnowModel-LG

The SnowModel-LG is a lagrangian model which provides daily snow on a 12.5 km grid over the Arctic^42^. The period covered runs from August 1st 1980 to July 31st 2021. Two versions are available using forcing from MERRA-2 or ERA5 and the sea ice concentration from NSIDC. OIB data are also assimilated. We use version 1 available at the following NSIDC access: https://daacdata.apps.nsidc.org/pub/DATASETS/nsidc0758_lagrangian_snow_distributions_v01/. We also converted this version to a monthly dataset on a 25 km grid.

### *In situ* and airborne data

Satellite altimetry provides synoptic data which must be validated against *in situ* measurements to assess their quality. As a remote and hard-to-reach territory, the Arctic Ocean lacks *in situ* data in terms of both spatial and temporal coverage. Table 1 lists all the data we have gathered over the period covered by IS2, since 2018. Note that apart from the automatic mooring BGEP (Beaufort Gyre Exploration Project) measurements, all *in situ* campaigns were suspended during the Covid period.

All datasets have been regridded with a 25 km resolution to be coherent with other datasets used in this study.

#### Operation IceBridge (OIB)

**Fig. 1:**
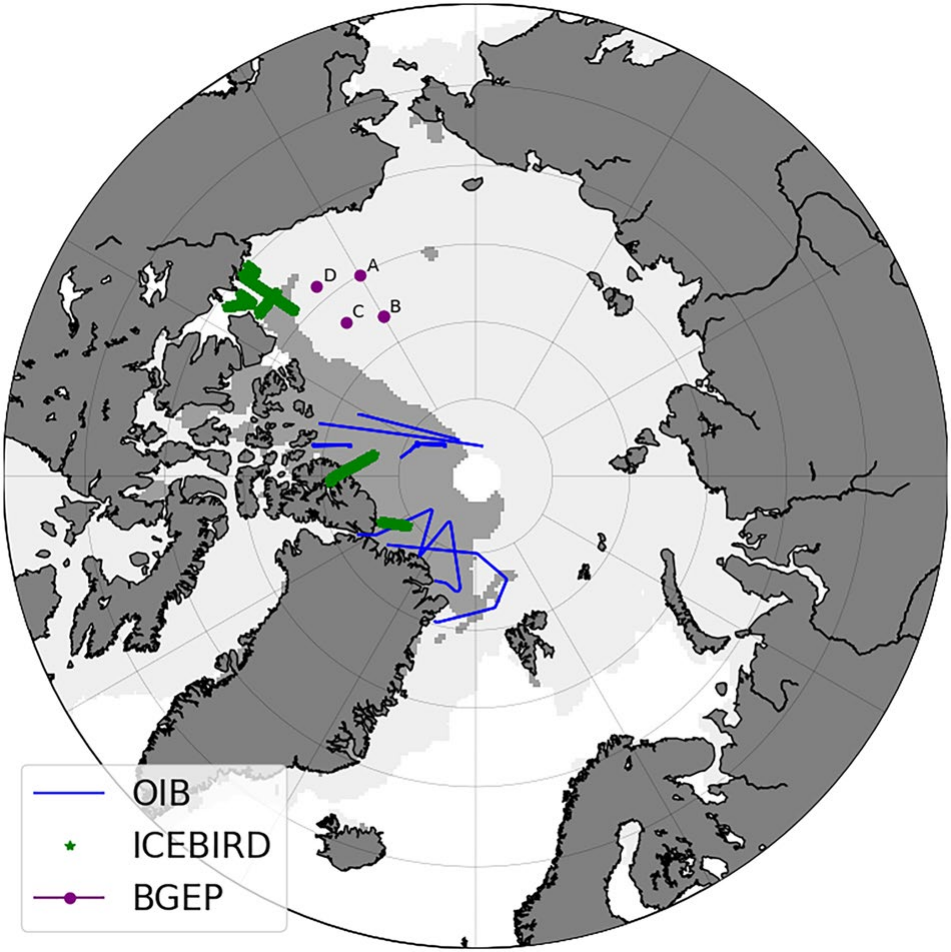
Map of the *in situ* data in the Arctic ocean used in this study: airborne total freeboard and snow depth with OIB, total ice thickness and snow depth with IceBird, sea ice draft with BGEP moorings.

Operation IceBridge (OIB) is a 10-years NASA’s effort aimed to collect polar data to fill the gap between the end of the ICESat mission in 2009 and the beginning of the IS2 mission in 2018, with a last campaign in 2019. It consists of airborne measurements (in blue on Fig. 1 during the April 2019 campaign) mainly over multi-year ice near the Canadian and Greenland coasts. In the Arctic the campaigns took place every year in March, April or May.

The aircraft hosted a variety of instruments including a Ku-band radar altimeter, a laser altimeter and a snow radar to characterize the sea ice thickness and the snow depth. Acquisitions were made at an average height of 1500 feet (457 m) which depending on weather conditions could vary between 1000 and 2000 feet (305 and 610 m) above the ground. We use the NSDIC product^43^ available at https://nsidc.org/data/nsidc-0708/versions/1. The spatial resolution is about 40 m between each measurement. We gridded all data to an EASE2 format with 25 km of resolution.

#### IceBird

The IceBird program is led by the Alfred Wegener Institute (AWI). It consists of airborne surveys, which took place once in summer and once in spring (March/April) almost every year since 2010. The aircraft trajectories for 2019 are shown in green on Fig. 1.

The aircraft carries an Electromagnetic Induction sounding instrument (EM-bird), used to measure the total sea ice thickness (ice + snow), a laser scanner, a camera providing photos of the sea ice surface and a snow radar to measure snow depth on sea ice surfaces. The plane is flying at about 60 m from the surface and the towed bird takes measurements about each 4-5 m. We used data from^44^ (https://doi.pangaea.de/10.1594/PANGAEA.932790) which has been gridded at 25 km resolution.

#### BGEP moorings

The Beaufort Gyre Exploration Project (BGEP) aims at monitoring the Beaufort Gyre basin to study fresh water and its variability using sensors moored below the ice. It started in summer 2003. Four moorings (A-D) have been deployed throughout the years, but mooring C has stopped producing data since 2008 (purple dots on Fig. 1). They provide a daily time series of temperature, salinity, currents, sea ice draft and bottom pressure. Sea ice draft is measured thanks to an upward-looking sonar mounted in the mooring at 50 m depth which sends a pulse reflected by the surface water or the sea ice.

The sea ice draft obtained with BGEP has an uncertainty of +/− 0.3 m.

We use daily data from https://www2.whoi.edu/site/beaufortgyre/data/mooring-data/, smoothed with a running mean of 6 days for the display to observe the variability and then average monthly for the statistics with the monthly altimetry products.

In the next sections, the comparisons will be done between the sea ice draft from BGEP and the draft derived from the snow depth products and a freeboard product (see the technical validation section).

## Methods

**Fig. 2:**
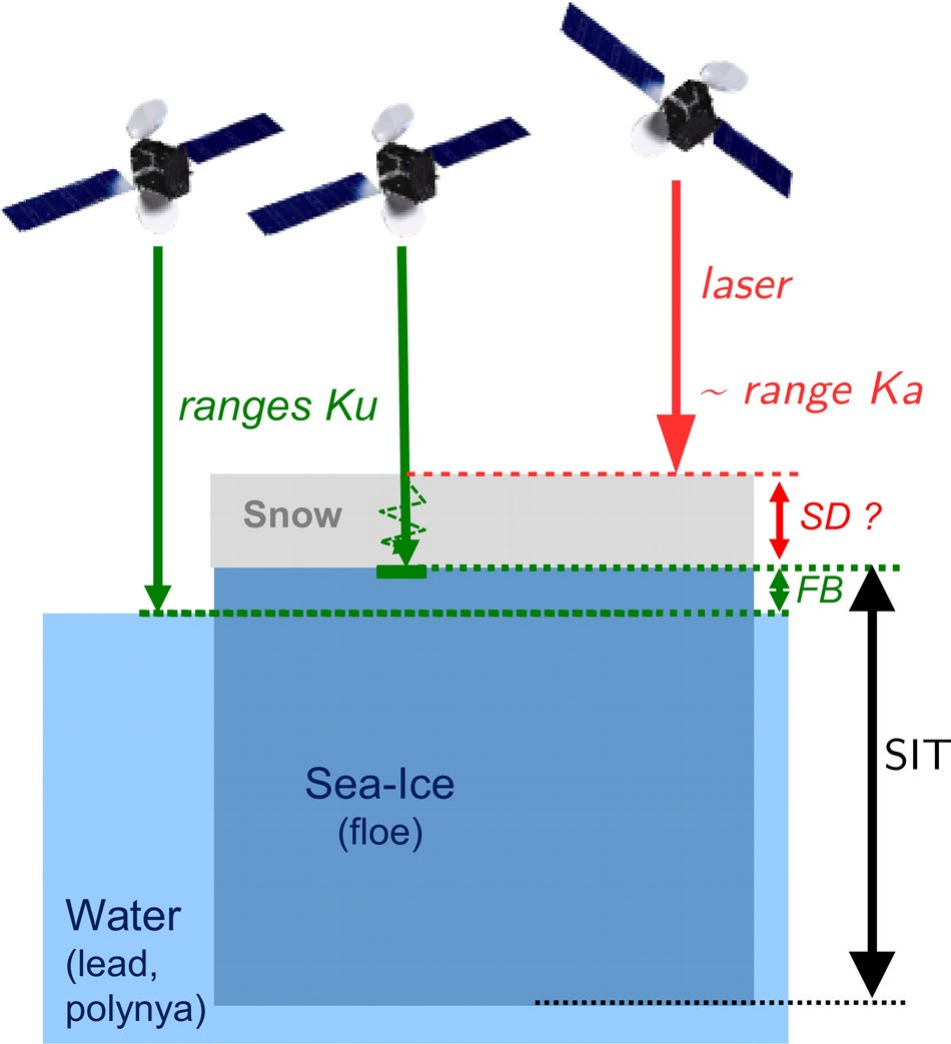
Scheme of the sea ice measurements principle from space.

Sea ice thickness is a difficult parameter to measure from space. According to its density, sea ice could have 60% to 90% of its volume underwater. The emerging part is often covered by a layer of snow that can reach several tens of centimetres. Satellite altimetry missions, such as CS2 and IS2 can measure the height of the emerging part, called the freeboard (FB on Fig. 2). From this freeboard, the thickness of the ice can be deduced from the hydrostatic equilibrium equation Equ. 1, provided that snow layer parameters (thickness *SD* and density *ρ*_*s*_) and the ice and water densities *ρ*_*i*_ and *ρ*_*w*_ are known.

1 $${SIT}=\frac{{\rho }_{w}}{{\rho }_{w}-{\rho }_{i}}{{FB}}_{{ice}}+\frac{{\rho }_{s}}{{\rho }_{w}-{\rho }_{i}}{SD}$$

where FB_ice_ is the ice freeboard.

Our current knowledge of the snow layer is poor, resulting in substantial errors. As it will be shown further in the paper, snow depth uncertainty is one of the main sources of uncertainty on the measure of sea ice thickness. In the same section we can observe that the other important sources of uncertainties on the sea ice thickness come from the density of the ice and the quality of the freeboard measurement, which uncertainties are probably of a magnitude similar to snow depth uncertainties but with less variability (more systematic errors).

The laser altimeter aboard IS2 can be reliably assumed to not penetrate the snow and provides a measure of the total freeboard, i.e., the ice freeboard plus the snow thickness (Equ. 2), and from Equ. 1 we can evaluate the *SIT* using Equ. 3:

2 $${{FB}}_{{ice}}={{FB}}_{{La}}-{SD}$$

where FB_La_ is the freeboard measured by laser altimetry assimilated to the total freeboard.

3 $${SIT}=\frac{{\rho }_{w}}{{\rho }_{w}-{\rho }_{i}}{{FB}}_{{La}}+\frac{{\rho }_{s}-{\rho }_{w}}{{\rho }_{w}-{\rho }_{i}}{SD}$$

When the snow is dry, cold and fresh, the Ku frequency can penetrate several metres into the snow, right up to the snow/ice transition. This ideal situation is not always encountered, particularly on young ice that is still salty, in the event of flooding, or at the start of the spring melt. In the absence of knowing the state of the snow, we assume that the Ku frequency penetrates to the snow/ice interface. However, the snow reduces the speed propagation of the radar wave *c*_*s*_ with a factor relative to the speed of light *c* that depends on the snow density^45,46^. Using the Ulaby relationship *c/c*_*s*_ = *(1* + *Tρ*_*s*_*)*^*1.5*^, with T equal to 0.00051, the *FB*_*ice*_ and the *SIT* can be expressed as:

4 $${{FB}}_{{ice}}={{FB}}_{{Ku}}+{SD}\left(\frac{c}{{c}_{s}}-1\right)$$

where FB_Ku_ is the freeboard measured by Ku-band altimetry.

5 $${SIT}=\frac{{\rho }_{w}}{{\rho }_{w}-{\rho }_{i}}{{FB}}_{{Ku}}+\frac{{\rho }_{w}{(1+T{\rho }_{s})}^{1.5}-{\rho }_{w}+{\rho }_{s}}{{\rho }_{w}-{\rho }_{i}}{SD}$$

If both Ku and Laser freeboards are measured, we can obtain an estimation of the snow depth *SD* by subtracting these two freeboards and taking into account a propagation factor (Equ. 6). If reliable, the knowledge of SD may substantially reduce the remaining uncertainty on *SIT* which can be expressed as a function of *FB*_*Ku*_ and *FB*_*La*_ (Equ. 7):

6 $${SD}=\left({{FB}}_{{La}}-{{FB}}_{{Ku}}\right)\frac{{c}_{s}}{c}$$

7 $${SIT}=\frac{{\rho }_{w}{{FB}}_{{La}}+\left({\rho }_{s}-{\rho }_{w}\right)\left({{FB}}_{{La}}-{{FB}}_{{Ku}}\right)/{\left(1+T{\rho }_{s}\right)}^{1.5}}{{\rho }_{w}-{\rho }_{i}}$$

However, the ability of the Ku-band to penetrate the snow depends on the characteristics of the snow (salinity, moisture, density, grain size) and its surface roughness^47^. The snow properties have been investigated by^19,48–50^. Under certain conditions, which remain to be better understood and characterised, the snow depth may therefore be underestimated. However, the error is limited by the stable upper bound of the IS2 total freeboard (FB_laser_ = FB_ice_ + SD): an underestimation of the snow depth is compensated by an equivalent overestimation of the ice freeboard and the error will be only related to the difference of density between the snow and the ice.

The freeboard estimates could also be impacted by processing methods such as the methodology to retrack the radar waveform or the classification routine.

To compute the LaKu LEGOS snow depth, we use both the TFMRA50 and the SAMOSA+ datasets described in the section about CS2. This leads to two snow depth estimates, one for each retracker. Both values are provided in the final product but in this study we only consider SAMOSA+ version because it provides a better agreement with OIB and ICEBird and comparable results for BGEP (details are given in the corresponding sections).

We chose to use a water density ρ_w_ of 1024 kg.m^−3^^22,51^. Concerning the snow density ρ_s_ the litterature mention 300 kg.m^-3^^22,52^ or the use of Mallett equation (SI CCI documents: https://climate.esa.int/media/documents/SeaIce_CCI_P1_ATBD-SIT_D2.1_Issue_3.1_signed.pdf) which is:

8 $${{\rm{\rho }}}_{{\rm{s}}}=6.50* {\rm{nbMonthFromOct}}+274.51$$

where nbMonthFromOct is the number of month since October.

We chose to use the lattest after a sensivity analysis.

The density of ice ρ_i_ is usually taken as 882 kg.m^−3^ over MYI and 917 kg.m^−3^ over FYI^40,53^. We chose here to use a value of 900 kg.m^−3^ as the average between FYI and MYI values. We believe that these values do not take into account the changes in salt that occur over a winter. It would be interesting to implement an ice density correction with an evolution from October to April but this is beyond the scope of this paper. We plan to further investigate the impact of the densities on the snow depth products in the future.

The LaKu snow depth product is provided as a set of monthly maps with a spatial resolution of 25 km which seems to be a good compromise between the best resolution possible and the technical limits of our products and the noise of the instruments.

Comparing to other LaKu products described in the data section the main differences consist in the datasets used (ATL20 gridded freeboard for LaKu UiT and LaKu UoL with different versions, ATL10 for LaKu Kacimi&Kwok and LaKu LEGOS), the retrackers used (LARM for UiT, SAMOSA+ for LEGOS, adaptive centroid for Kacimi&Kwok and threshold for UoL), the methods (calibration of the freeboards using OIB data for LaKu UoL, maximum delay of 10 days and spatial distance of 75 km for Kacimi&Kwok) and the spatial resolution (50 km for UiT, 25 km for LEGOS and Kacimi&Kwok, 26 km for UoL).

## Data Records

The database is deposited on Zenodo under 10.5281/zenodo.14000765^54^. This monthly product covers 3 winters from October to April. Each netcdf file is named SD_LaKu_LEGOS_NH_yyyymm.nc where *yyyy* represents the year and *mm* the month. It covers latitudes higher than 50°N with a spatial resolution of 25 km. Each file contains:

- coordinates: latitude, longitude
- freeboard variables: freeboard_ku_sam (freeboard obtained from CryoSat-2 using SAMOSA+ retracker), freeboard_ku_t50 (freeboard obtained from CryoSat-2 using TFMRA50 retracker), laser_fb, freeboard_ka_t50 (freeboard obtained from SARAL using TFMRA50 retracker)
- snow depth variables: snow_depth_laku_sam, snow_depth_laku_t50, snow_depth_kaku_t50, sd_plrm_kaku, sd_plrm_laku
- standard deviation of freeboards and SLA: freeboard_ku_sam_std, sla_sam_std, freeboard_ku_t50_std, sla_t50_std, laser_fb_std
- uncertainties: SD_sam_uncertainties, SD_t50_uncertainties, SD_kaku_uncertainties
- additional parameters: icetype, snow_density, sea_ice_thickness, sea_ice_draft, sla_sam, sla_t50, delta_fb_plrm, gaussian_w, sla_swath

All these variables are described in the netcdf files.

## Technical Validation

In this section we show comparisons between all the products described. We first focus on the most similar products from satellite altimetry, then our snow depth product is compared to other snow depth products such as W99, PIOMAS or AMSR and finally to *in situ* measurements.

### Comparison to other satellite altimetry snow depth products

**Fig. 3:**
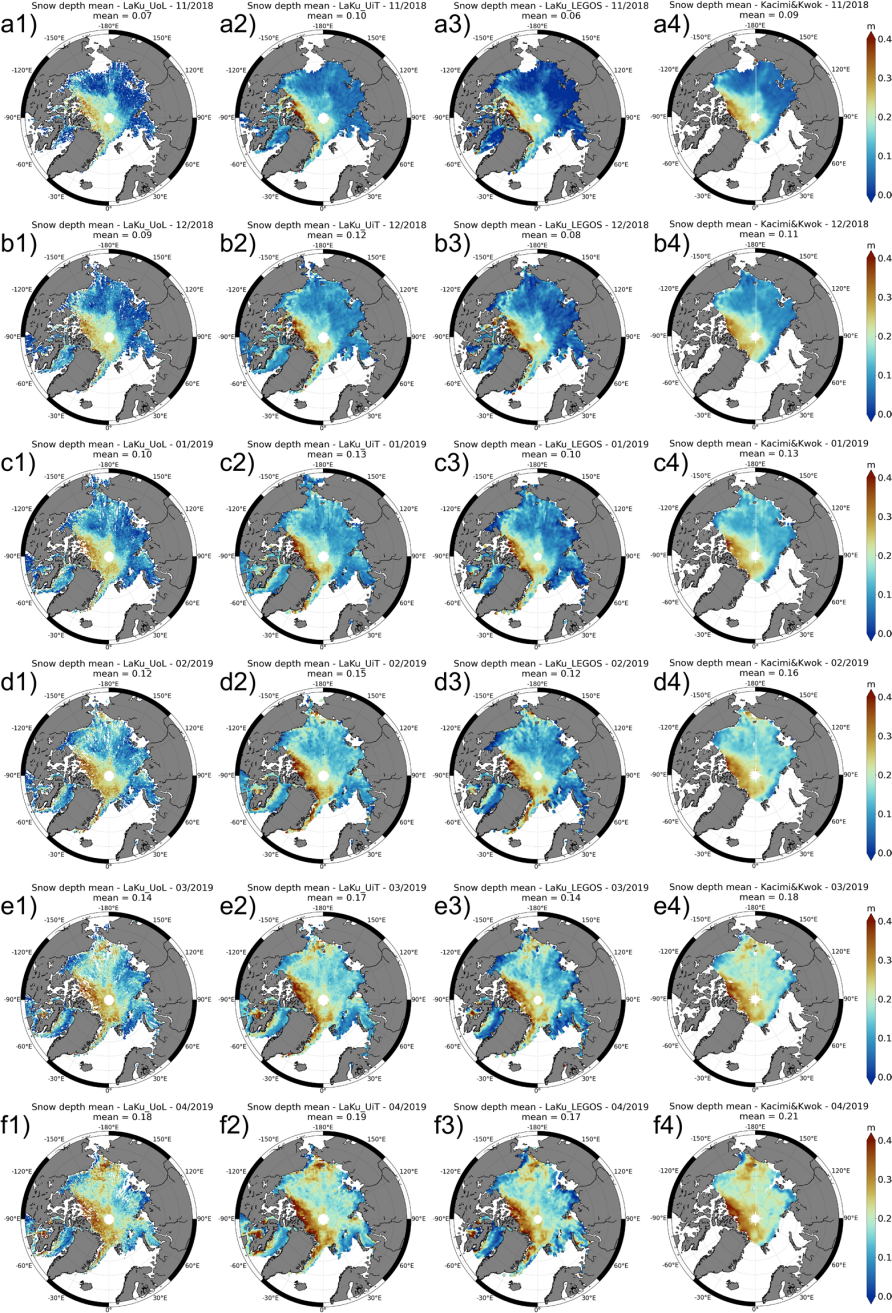
Maps of the monthly snow depth over (**a**) 11/2018; (**b**) 12/2018; (**c**) 01/2019; (**d**) 02/2019; (**e**) 03/2019; (**f**) 04/2019 for LaKu from (1) UoL; (2) UiT; (3) LEGOS; (4) Kacimi&Kwok.

**Fig. 4:**
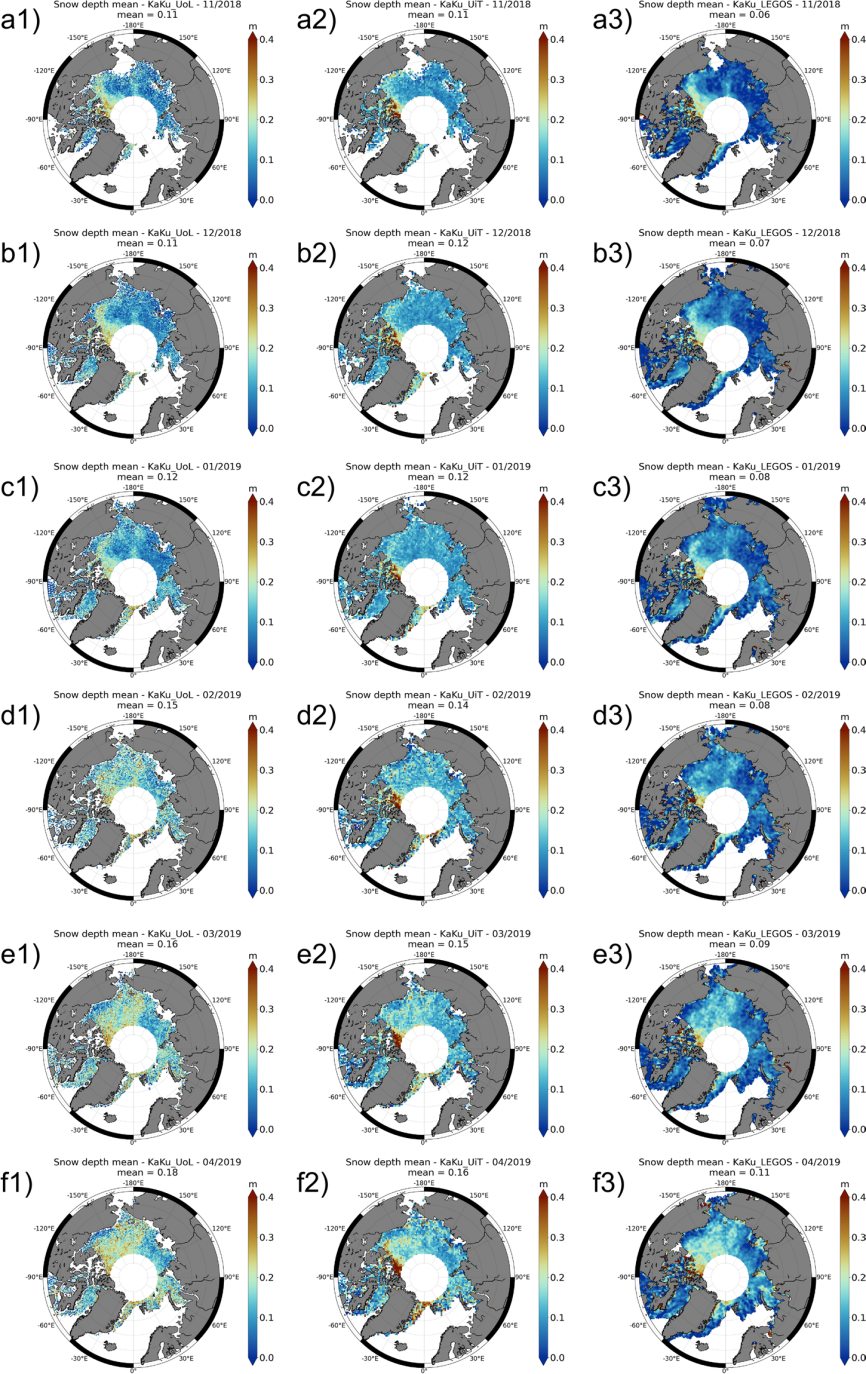
Maps of the monthly snow depth over (**a**) 11/2018; (**b**) 12/2018; (**c**) 01/2019; (**d**) 02/2019; (**e**) 03/2019; (**f**) 04/2019 for KaKu from (1) UoL; (2) UiT; (3) LEGOS.

We first compare the LaKu (Fig. 3) and KaKu (Fig. 4) altimetry snow depth products.

The KaKu and LaKu products don’t have the same spatial coverage as SARAL does not cover latitudes above 81.5°. The use of IS2 enables to obtain snow depth at higher latitudes.

Figures 3, 4 show the temporal and spatial variations of the snow depth over one winter (from November 2018 to April 2019) for the LaKu and KaKu products, respectively.

For the winter 2018–2019 we compute the variation of the amplitude of the mean snow depth between April and November. A common monthly mask is applied to all products. The observed average snow depth increase from November 2018 to April 2019 is different between products. It is higher for the LaKu products (0.11 m, 0.09 m, 0.11 m and 0.12 m for the UoL, UiT, LEGOS and Kacimi&Kwok products, respectively) than for the KaKu products (0.07 m, 0.05 m and 0.06 m for the UoL, UiT and LEGOS products, respectively). First we note that the LaKu products are consistent with each other, as are the KaKu products. From Figs. 3, 4 we observe that the KaKu products change little in early winter unlike the LaKu products which change more regularly and significantly. To further investigate this point we have plotted the evolution of UiT and LEGOS Ka, Ku and Laser freeboards over this winter (not shown). The laser freeboards are very consistent with a bias of only about 0.01 m which shows small differences between ATL20 version 3 and ATL10 version 5. The freeboard measured with Ka-band progresses much more slowly than the freeboard measured with the laser. LEGOS and UiT Ku SAR freeboards, both computed using a physical retracker, show similar patterns although with a significant bias of 0.06 m. The Ku and Ka freeboards of the KaKu LEGOS product have been computed from (pseudo)LRM waveforms using the TFMRA50 retracker and show significant differences. It could come from surface roughness effects for which LRM is very sensitive, but even more probably from under-estimation of penetration capabilities of the Ka-band within the snow. Further investigation is needed for future work. An analysis between laser and Ka frequencies should also be extended to the SWOT mission to determine whether it has the same effects on snow as Saral, despite its very different acquisition mode (swath).

Table 2 shows the R correlations between the snow depth products.

**Table 2:** The correlations (at top right of the diagonal) and mean bias (at bottom left of the diagonal) between the altimetric snow depth products over the common period: 10/2018-04/2019, 10/2019-04/2020, 10/2020-04/2021.

correlation R between products		LaKu				KaKu		
Mean bias in m (column - row)		UoL	UiT	LEGOS	Kacimi&Kwok	UoL	UiT	LEGOS
**LaKu**	**UoL**	—	0.83	0.83	0.87	0.49	0.32	0.55
	**UiT**	0.02	—	0.89	0.94	0.34	0.32	0.41
	**LEGOS**	−0.01	−0.02	—	0.94	0.37	0.35	0.47
	**Kacimi&Kwok**	0.02	0.00	0.03	—	0.45	0.51	0.71
**KaKu**	**UoL**	0.03	0.00	0.03	0.00	—	0.34	0.40
	**UiT**	0.01	−0.01	0.02	−0.01	−0.01	—	0.30
	**LEGOS**	−0.04	−0.06	−0.03	−0.06	−0.07	−0.06	—

The four LaKu snow depth products correlate very well with each other (correlations from 0.83 to 0.94) which shows good stability of this approach. The monthly means are also close to each other and vary from 0.07 to 0.18 for LaKu UoL, 0.10 to 019 for LaKu UiT, 0.06 to 0.17 for LaKu LEGOS and 0.09 to 0.21 for Kacimi&Kwok. The best correlation (0.94) is obtained between the Kacimi&Kwok and LEGOS / UiT products although they do not all use the same IS2 product (ATL10 v5 for Kacimi&Kwok, ATL10 v6 for LEGOS and ATL20 v2 for UiT). Thus the IS2 product sems to have little impact on the results. On the other hand Laku UoL has a lower correlation with the other LaKu products (see Table 2). The main difference comes from the computation of the CS2 freeboard wich does not account for the surface roughness for LaKu UoL (threshold for UoL, physical for UiT and LEGOS and adaptive for Kacimi&Kwok). The choice of the CS2 retracker seems to have a predominant effect on the results.

In contrast, KaKu products show a low correlation with each other (from 0.30 to 0.40), as well as with LaKu products (from 0.32 to 0.55). There are also more differences for the monthly means which vary from 0.11 to 0.18 for KaKu UoL, 0.11 to 0.16 for KaKu UiT and 0.06 to 0.11 for KaKu LEGOS. This might be due to the very different approach used by the three KaKu products: LEGOS compares Saral’s LRM with CS2’s pLRM and uses the TFMRA50 retracker, while both UiT and UoL use CS2’s SAR data retracked with the LARM physical retracker, for UiT, and a heuristic retracker for UoL. Slightly more significant correlations, however, seem to be obtained by the LEGOS product, exploiting CS2’s pLRM, reinforcing the hypothesis of the importance of having comparable radar footprint sizes to minimise the impact of different observed surface roughness^16^.

### Comparison to other snow depth products

This section focuses on the snow depth product obtained by methods other than altimetry. The five considered products are AMSR-2, PIOMAS, W99, NESOSIM and SnowModel-LG.

For each month of the common period which covers 11/2018-04/2019, 11/2019-04/2020 and 11/2020-04/2021 a common spatial mask has been applied to all datasets. Then each product is time-averaged over all the months of the 3 winters before we compute the statistics (correlation, RMSD compared to a reference and standard deviation of each single product).

**Fig. 5:**
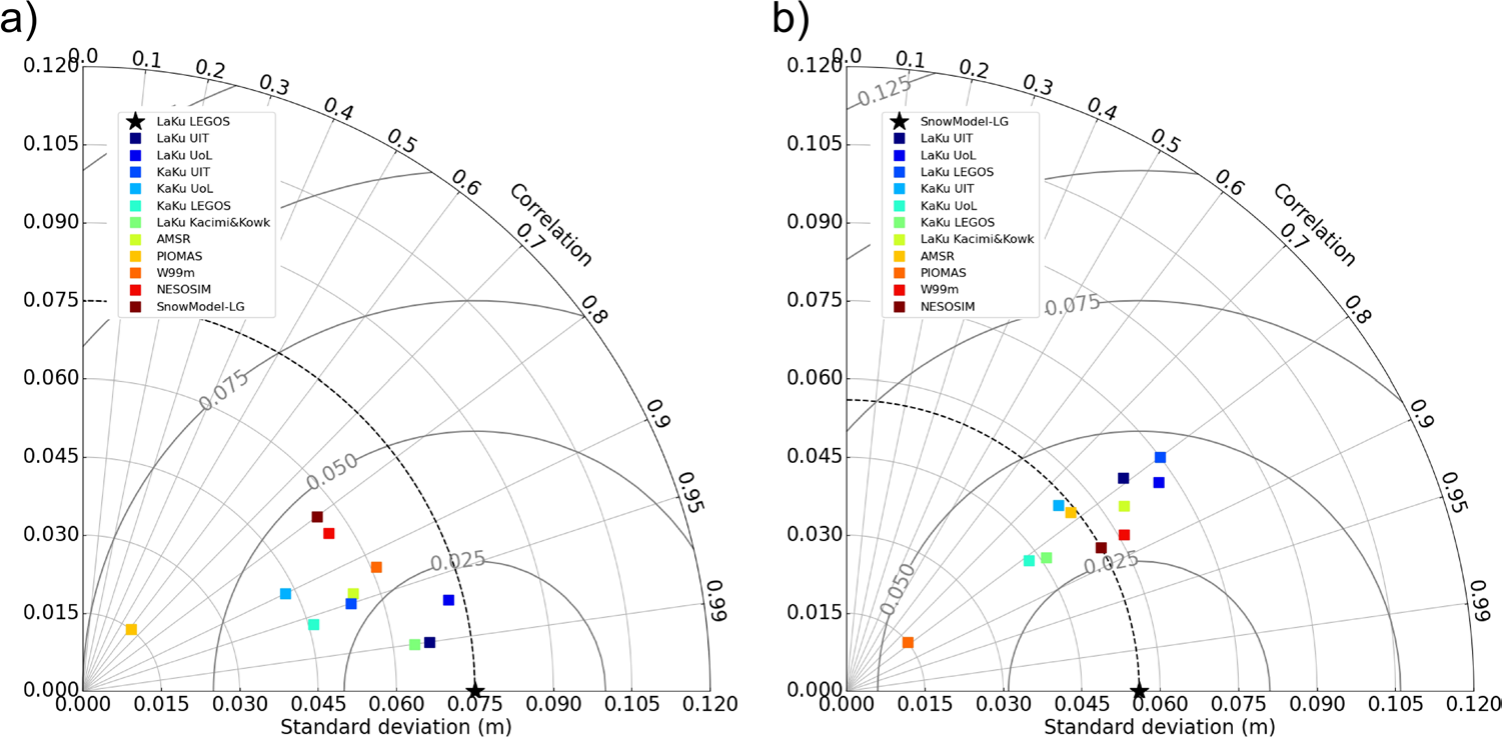
Taylor Diagrams with (**a**) the LaKu LEGOS product and (**b**) the SnowModel-LG product as a reference (black star at the bottom).

These statistics have been gathered in the two Taylor diagrams (Fig. 5). Taylor diagrams are used to assess the degree of correspondence between 2 products through 3 statistics: correlation, RMSE and standard deviation. The standard deviation is indicated on the x and y-axes with a black star for the reference; the correlation corresponds to the azimuthal angle and the RMSD to distance from the black star on the x-axis. These two diagrams only differ by the used reference which is LaKu LEGOS product on the left (a) and the SnowModel-LG on the right (b). We use both references to provide the statistics as a function of our product and as a function of an independent widely used model.

First we can observe that the correlations with the different products are all above 0.8 with LaKu LEGOS except for PIOMAS and above 0.75 with SnowModel-LG. LaKu LEGOS shows particularly good correlation with LaKu_UiT (0.99) and Kacimi&Kwok (0.98). Relative to SnowModel-LG, the best correlations are obtained with the other model, NESOSIM (0.84) and with W99m (0.87).

**Fig. 6:**
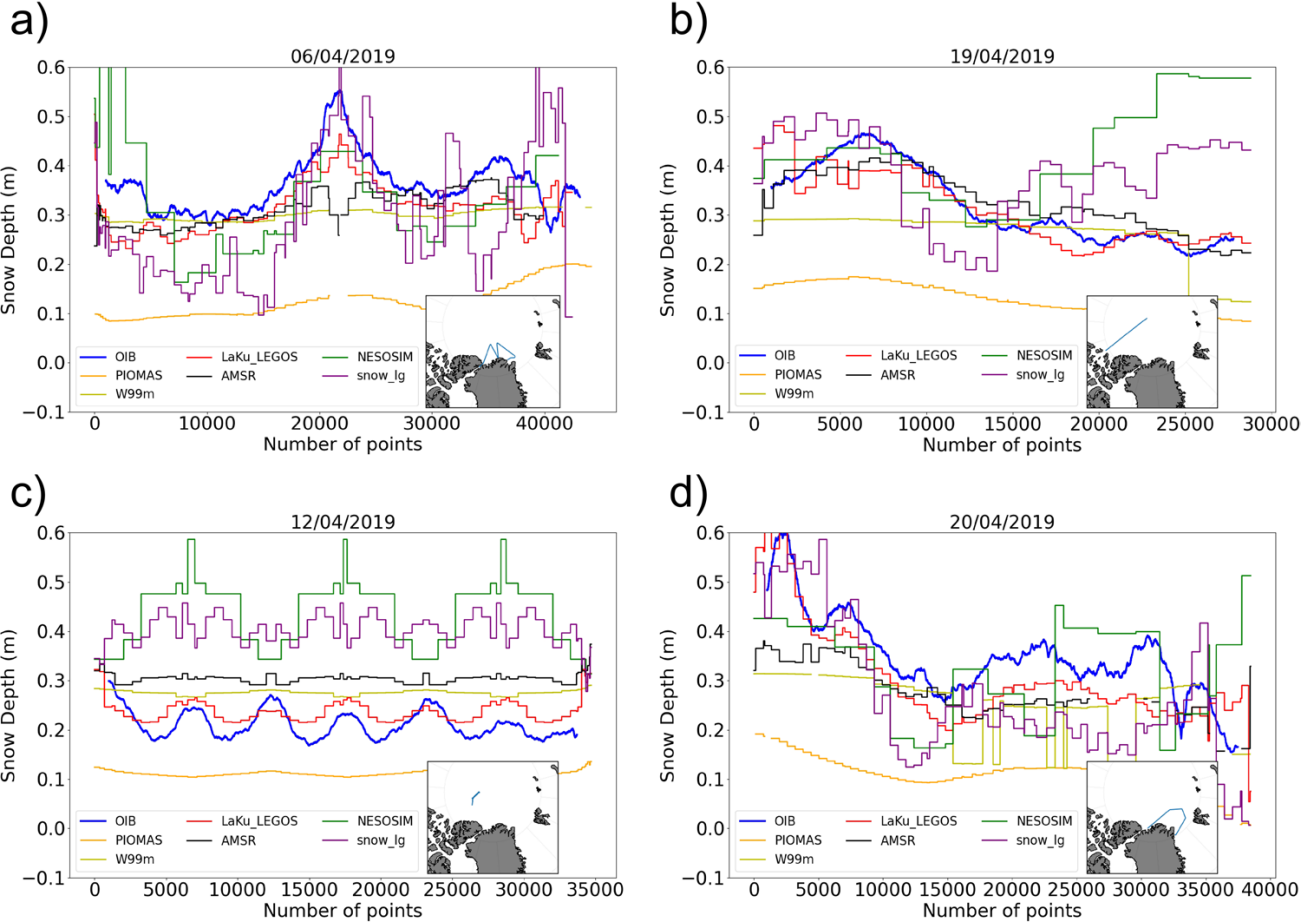
Snow depth along OIB flight tracks. Each panel corresponds to one flight. OIB snow depth is in blue, LaKu LEGOS in orange, AMSR-2 Bremen in black, SnowModel-LG in green, NESOSIM in purple, W99m in khaki and PIOMAS in orange.

The low standard deviation shown by PIOMAS (0.015) indicates this product misses fine scale processes. The reference standard deviations are 0.075 m for LaKu LEGOS and 0.056 m for SnowModel-LG. A lower standard deviation would have been expected for W99m as it is the case for W99. The correction applied on FYI leads however to strong discontinuities between FYI and MYI (visible on Fig. 6b,d) which increases the standard deviation.

Finally the RMSD are the lowest between LaKu_UoL and UiT and LaKu LEGOS (0.022 m) and between NESOSIM and SnowModel-LG (0.029 m). This is not surprising, as the respective approaches are similar, using altimetry on the one hand and reanalysis on the other.

### Comparison to *in situ* products

In this section, we compare satellite and model snow depth products with available *in situ* data. We highlight the importance of *in situ* reference measurements for validation and calibration purposes and the necessity of additional airborne campaigns and fieldwork to accurately evaluate the added value of each product (altimetry,models, space radiometry). In particular snow depth data are drastically lacking throughout the seasons in the Arctic (OIB program is over since 2019 and ICEBird is not regular).

#### Comparisons with airborne snow depth from OIB

The last OIB campaign took place in 2019. It is the only one which coincides with IS2. Figure 6 shows four flights along which the snow depth has been measured on the 6th, 12th, 19th and 20th of April 2019. Two other flights took place on the 8th and 22nd of April. We also represented the scatter plots of each product against OIB for all the tracks (not shown) and computed the statistics. A good agreement with the LaKu LEGOS product can be observed with a correlation of 0.72 for all the tracks. The W99m product does not show the spatial variations of the snow depth along the tracks (correlation of 0.36 with the gridded product containing all the tracks). The AMSR product is able to reproduce some spatial variations but not for scales as fine as for the LaKu LEGOS product (correlation of 0.50 but the slope is 0.30). The PIOMAS product is also underestimating the snow depth in comparison to OIB although large scale variations are reproduced. NESOSIM and SnowModel-LG cannot reproduce snow depth variations at the small scales observed by OIB.

**Fig. 7:**
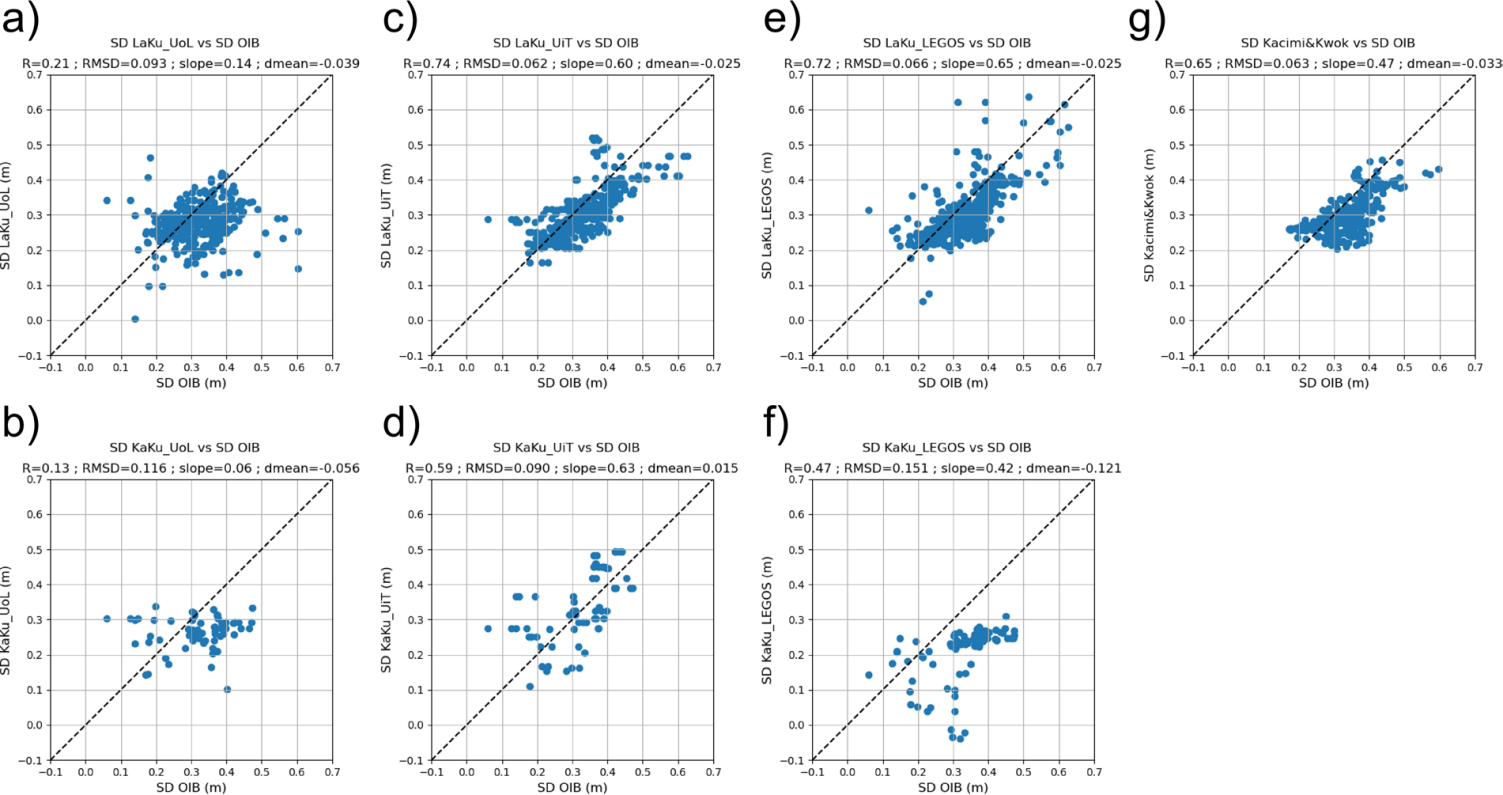
Scatter plots of OIB data vs LaKu products from (**a**) UoL; (**c**) UiT; (**e**) LEGOS (**g**) Kacimi&Kwok and KaKu products from (**b**) UoL; (**d**) UiT; (**f**) LEGOS. The statistics are given in the title of each sub panel, where R stands for the correlation coefficient, RMSD for the Root Mean Square Differences and mean for the mean bias.

We have also compared the different LaKu and KaKu snow depth solutions against the OIB gridded product. Figure 7 displays the scatter plots of the monthly gridded OIB snow depth measurements for April 2019 versus each of these products.

The reduced number of measurements for the KaKu scatters are due to the SARAL spatial coverage limited to 81.5°N and therefore only partially overlapping with the OIB flights. Some negative values can be seen in Fig 7. This is mainly due to the fact that the orbits of the two satellites are different and, as a result, they do not not acquire at the same date data from a given location. When the two freeboards are close in time, noise effects on the measurements can also lead to negative differences. In order to be able to carry out static analyses, we have chosen to keep all the measurements, but if this product is to be used, for example, to calculate the thickness of the ice from freeboard measurements, we recommend setting the negative values to zero. The best correlations with OIB snow depth measurements are obtained here for LaKu UiT (0.74), LaKu LEGOS (0.72) and LaKu Kacimi&Kwok (0.65). For these 3 products, the biases are limited to 0.033 m. They also capture the dynamic range of the snow depth, with a slope closer to 1 than other products (even if they do not capture the full spread, with slopes up to 0.65 for LaKu LEGOS). LaKu UoL and KaKu LEGOS and UoL products have a lower R and higher RMSD, with a lower precision reproducing the airborne snow depths. Of the non-altimeter products, AMSR and W99m best reproduce the airborne observations, with RMSD of 0.07 and 0.09 m respectively and a bias of a few cm, but with slopes of 0.30 and 0.22. The reanalysis- and model-based products generally do not capture the variability of the airborne snow depths, with RMSD from 0.13 (SnowModel-LG) to 0.21 (PIOMAS) m and lower correlations. PIOMAS snow depths underestimate the airborne data by 20 cm. Readers should bear in mind, however, that these statistics which are summarised in Table 3 are for a single month only.

**Table 3:** Comparisons between airborne gridded OIB snow depth measurements and the gridded products during April 2019.

OIB versus		R	RMSD	slope	Bias
**LaKu**	**UoL**	0.21	0.09	0.14	−0.04
	**UiT**	0.74	0.06	0.60	−0.02
	**LEGOS**	0.72	0.07	0.65	−0.02
	**Kacimi&Kwok**	0.65	0.06	0.47	−0.03
**KaKu**	**UoL**	0.13	0.12	0.06	−0.06
	**UiT**	0.59	0.09	0.63	0.02
	**LEGOS**	0.47	0.15	0.42	−0.12
**non altimetry products**	**AMSR**	0.50	0.07	0.30	−0.02
	**PIOMAS**	0.57	0.21	0.23	−0.20
	**W99m**	0.36	0.09	0.22	−0.05
	**NESOSIM**	−0.04	0.14	−0.05	0.03
	**SnowModel LG**	0.29	0.13	0.42	−0.01

Concerning the LEGOS LaKu solution these results are provided using the SAMOSA+ physical retracker as described in section Source Data. If we use the TFMRA50 empirical retracker (not shown) the correlation obtained with OIB is 0.40 so rather lower than with SAMOSA+. Empirical retracker are much easier to implement than physical retracker but the latest has already shown to provide more accurate measurements^33,55^. Indeed they are based on waveform model fitting which allows to take into account the scattering properties and the multi-scale surface roughness. Physical retrackers capture more realistic variations in the properties of the ice. On the contrary threshold retrackers do not take into account the large scattering variabilities from leads to floes which significantly impact the range retrieval^27^.

#### ICEBird

**Fig. 8:**
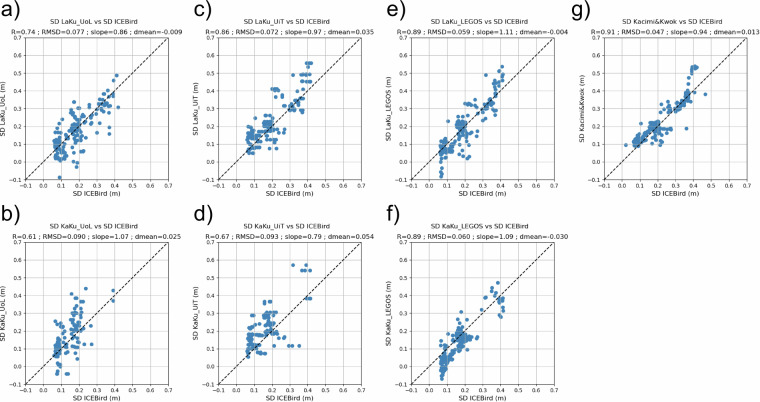
Scatter plots of ICEBird snow depth vs LaKu products from (**a**) UoL; (**c**) UiT; (**e**) LEGOS; (**g**) Kacimi&Kwok and KaKu products from (**b**) UoL; (**d**) UiT; (**f**) LEGOS over 04/2019. The statistics are given in the title. R stands for the correlation coefficient, RMSD for the Root Mean Square Differences and mean for the mean bias.

ICEBird tracks are located closer to the Canadian coast than OIB tracks, which go from the coast to higher latitudes (Fig. 1). As for OIB, the ICEBird tracks from April 2019 have been gridded and the mean snow depth value obtained for each pixel is compared with the different altimetric snow depth measurements in the scatter plots in Fig. 8.

With variations from 0.61 to 0.91, the altimetry snow depth products are better correlated to the ICEBird data than to the OIB data (0.13 to 0.74). The best correlations are obtained for the LaKu Kacimi&Kwok (0.91), LaKu LEGOS (0.89) and KaKu LEGOS (0.89) products. The mean biases are quite low ranging from -0.009 m (LaKu LEGOS) to 0.054 m (KaKu UiT). Using the TFMRA50 empirical retracker for the LEGOS product (not shown) leads to lower correlations (0.71).

#### BGEP moorings

On the contrary to ICEBird and OIB, BGEP moorings do not directly measure the snow depth. Thanks to their upward looking sonar the moorings provide the draft of the sea ice which by definition is equal to *SIT – FB*_*ice*_. Using this relationship and the Eq. 5 we can convert any pair (FB_Ku_, SD) to a draft.

In the following, we compare different snow depth products combined with a common freeboard product that is not associated with any of the presented SD products, the LEGOS TFMRA50 solution.

**Fig. 9:**
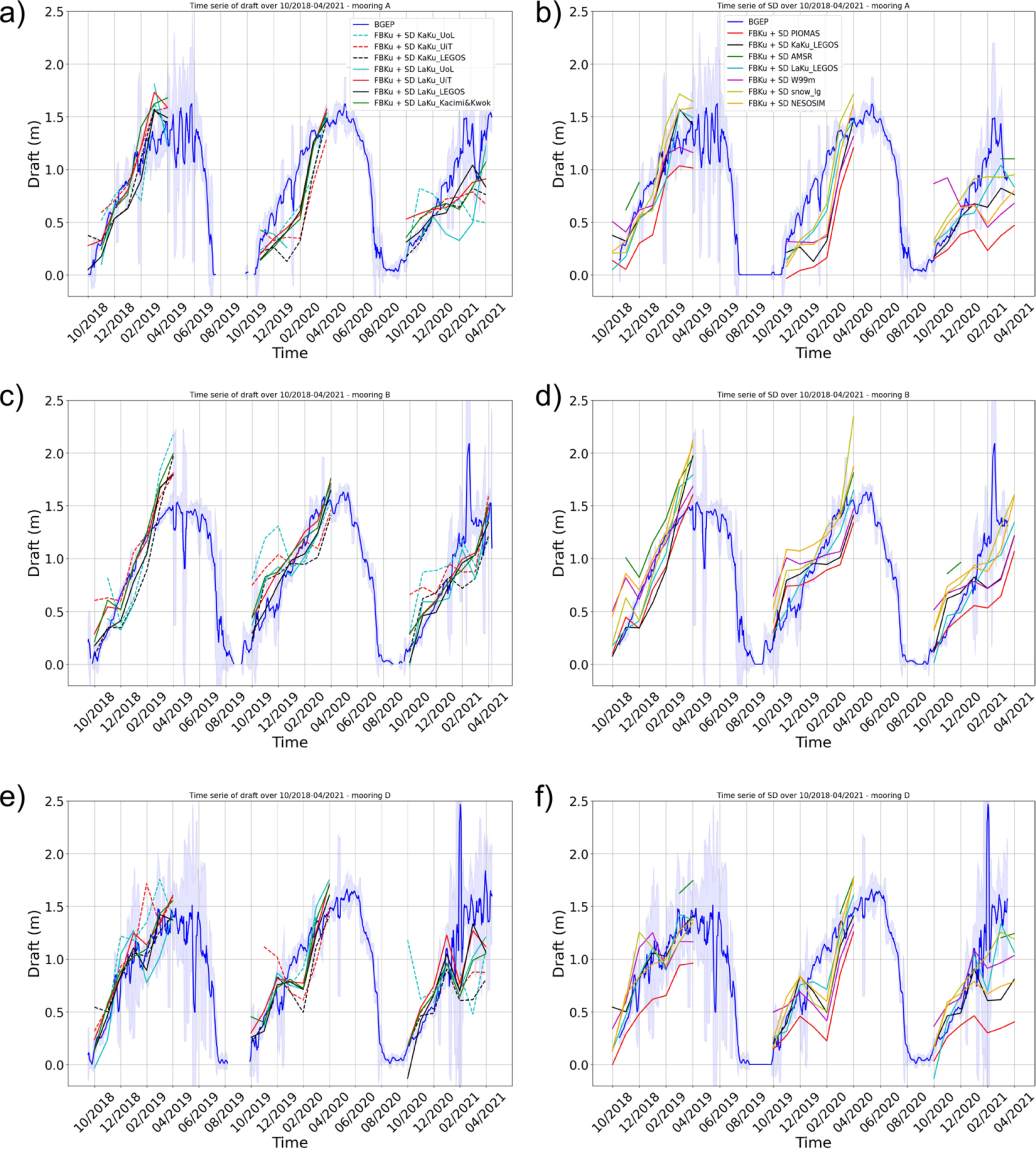
Time series of the draft from daily BGEP measurements (in blue) for (**a,b**) mooring A; (**c,d**) mooring B; (**e,f**) mooring D and of the draft deduced from altimetry snow depth products (first column), or LaKu and KaKu LEGOS products, AMSR, PIOMAS, W99m, NESOSIM and Snow-LG (second column), both combined with a CS2 TFMRA50 based radar freeboard to compute the draft equivalent parameter.

Each snow depth product was projected on the closest grid cell of the moorings location. The draft time series is represented on Fig. 9 for each of the 3 moorings. Plots 9a, c and e represent the time series for the altimetry snow depth products and plots 9b, d and f for other snow depth products. Our LaKu product is represented on all plots.

Overall the altimetry snow depth products provide drafts which are quite close to each other (Fig. 9a,c,e) with some exceptions at the beginning of winters and over winter 2020-2021 on the contrary to other products (W99m, PIOMAS, AMSR, SnowModel-LG and NESOSIM) which show a greater dispersion (Fig. 9b,d,f). Even if the draft is significantly driven by the chosen freeboard, the snow depth has a non-negligible impact on the results. When all solutions show a similar pattern variation (e.g. the drop in November 2019 at mooring D) this means that this variation is driven by the freeboard. In contrast when they present diverging variations it means that these variations are driven by the snow depth solutions (e.g. the drop in February 2019 at mooring D). For a common freeboard, an overestimation of the draft corresponds to an overestimation of the snow depth.

The drafts obtained from exclusively altimetry data, and especially the LaKu products, match quite well with the drafts from BGEP especially for winter 2018-2019 and winter 2019-2020. Concerning the drafts computed with snow depths from non altimetry products they are slightly thicker than the drafts from BGEP, apart from PIOMAS.

Table 4 gives the statistics between the monthly draft of BGEP and the monthly draft derived from each snow depth product. The LaKu products and the LEGOS one in particular have the best statistics compared to BGEP (correlations greater than 0.81, RMSD less than 0.31). One should bear in mind that the computation of draft also depends on the freeboard chosen, thus choosing a LEGOS solution is probably an advantage, even if it is not the same retracker (TFMRA50 instead of SAMOSA+), because combining similar products could help to compensate some errors. For instance, if a step of the processing like the retracking or waveform classification produces freeboards with a thick bias, then the derived snow depth would be biased thin, compensating slightly with a systematic offset.

**Table 4:** Comparative statistics between the monthly draft of BGEP and the monthly draft derived from each snow depth product over the common period: 10/2018-04/2019, 10/2019-04/2020, 10/2020-04/2021.

BGEP moorings versus		Correlation	RMSD	Slope	Bias
**LaKu**	**UoL**	0.81	0.31	0.89	−0.09
	**UiT**	0.85	0.24	0.84	0.01
	**LEGOS**	0.89	0.23	0.92	−0.08
	**Kacimi&Kwok**	0.83	0.26	0.87	−0.01
**KaKu**	**UoL**	0.55	0.41	0.54	0.02
	**UiT**	0.62	0.36	0.54	0.01
	**LEGOS**	0.76	0.33	0.71	−0.13
**non altimetry products**	**AMSR**	0.80	0.34	0.68	0.21
	**PIOMAS**	0.70	0.48	0.59	−0.35
	**W99**	0.63	0.35	0.46	−0.06
	**NESOSIM**	0.71	0.34	0.73	−0.01
	**SnowModel LG**	0.83	0.28	0.92	0.04

While LaKu solutions have a slope close to 1 with BGEP, KaKu solutions have lower slopes (between 0.54 and 0.71). This means that they observe snow depth growth poorly, as already observed from Figs. 3 and 4.

For these BGEP comparisons we use a freeboard obtained with the TFMRA50 retracker whereas the snow is obtained from the SAMOSA+ retracker. Higher statistics would have been expected by combining a freeboard and a snow depth product from the same retracker (eg SAMOSA+), which could compensate for some of the measurement errors, but this is not the case as the mean bias is largely reduced by using the TFMRA50 freeboard for all products (not shown). The reasons behind still need to be investigated but are beyond the scope of this paper.

**Fig. 10:**
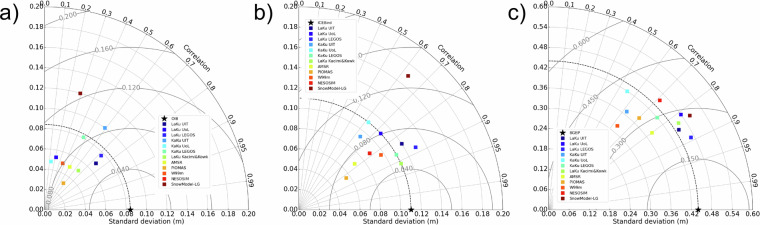
Taylor diagrams of all the snow depth products used in this study with reference to (**a**) OIB; (**b**) ICEBird; (**c**) BGEP.

To summarise the different diagnostics realised in this paper Fig. 10 shows the Taylor diagrams with each *in situ* product as a reference. Except KaKu UiT and SnowModel-LG all products have a smaller STD than OIB with KaKu and LaKu LEGOS being very similar (Fig. 10a). The correlation of all products with OIB is smaller than with BGEP (Fig. 10c) or ICEBird (Fig. 10b). KaKu and LaKu UoL, KaKu LEGOS and LaKu Kacimi&Kwok have a standard deviation almost identical to ICEBird whereas the closer STD value for BGEP is PIOMAS with most of the products having a greater STD than BGEP. The LaKu LEGOS product has the first or the second better correlation for each *in situ* product. The RMSD are higher for BGEP moorings because they refer to the draft and not the snow depth. In general the RMSD are the highest for SnowModel-LG and KaKu UoL and the smallest for LaKu UiT, LaKu LEGOS and Kacimi&Kwok.

The combination of Laser and Ku-band altimetry seems to provide currently the best snow depth estimations, more consistent relative to *in situ* measurements than the KaKu combination. In particular, LaKu LEGOS product compares quite well with *in situ* and airborne data. The differences that persist can be attributed to other factors such as the uncertainties linked to the level of penetration of the radar waves into the snow, the effects of the surface roughness, the footprint size of IS2 and CS2, the mode of acquisition (LRM, SAR, SARIN) and the processing methodology (retracker). We think that the greatest uncertainties come from the level of penetration in the snow however there is a drastic lack of *in situ* snow depth data which prevent further investigation.

These results provide evidence that significant Ku backscatter is returned to the radar from the snow-ice interface, producing radar freeboards that give relatively unbiased snow depths when combined with more certain laser freeboards. It is therefore reasonable to assume that the radar freeboard represents the sea ice freeboard, after accounting for the delayed wave propagation through snow, if there is not auxiliary information available on the expected penetration of the Ku-band radar for specific snow and sea ice conditions.

### Uncertainties

The knowledge of snow depth is of critical importance to retrieve sea ice thickness^56^ reported that the largest contribution to the error in sea ice thickness is due to the uncertainty in snow depth. In this section we focus on the impact of snow depth uncertainties on sea ice thickness uncertainties to investigate how it can be reduced.

As a first step in this analysis, let’s consider the case where snow depth is an auxiliary product derived from independent observations (eg, Warren 99, AMSR), a model (eg, NESOSIM, SnowModelLG, etc.), or any other source. With the additional assumption of a Gaussian distribution the sea ice thickness uncertainties can be computed from partial derivation of Equ. 3:

9 $$\begin{array}{c}{u}_{{SIT}}^{2}={u}_{{{FB}}_{{La}}}^{2}{\left[\frac{{\rho }_{w}}{{\rho }_{w}-{\rho }_{i}}\right]}^{2}+{u}_{{SD}}^{2}{\left[\frac{{\rho }_{s}-{\rho }_{w}}{{\rho }_{w}-{\rho }_{i}}\right]}^{2}+{u}_{{\rho }_{s}}^{2}{\left[\frac{{SD}}{{\rho }_{w}-{\rho }_{i}}\right]}^{2}+\\ {u}_{{\rho }_{w}}^{2}{\left[\frac{\left(-{\rho }_{i}{{FB}}_{{La}}+{SD}\left({\rho }_{i}-{\rho }_{s}\right)\right)}{{\left({\rho }_{w}-{\rho }_{i}\right)}^{2}}\right]}^{2}+{u}_{{\rho }_{i}}^{2}{\left[\frac{\left({\rho }_{w}{{FB}}_{{La}}+{SD}\left({\rho }_{s}-{\rho }_{w}\right)\right)}{{\left({\rho }_{w}-{\rho }_{i}\right)}^{2}}\right]}^{2}\end{array}$$

where u_parameter_ corresponds to the uncertainty of each of the input parameters.

Equation 9 can be rewritten with the following form:

10 $${u}_{{SIT}}^{2}={c}_{{{FB}}_{{La}}}^{2}{u}_{{{FB}}_{{La}}}^{2}+{c}_{{SD}}^{2}{u}_{{SD}}^{2}+{c}_{{\rho }_{s}}^{2}{u}_{{\rho }_{s}}^{2}+{c}_{{\rho }_{i}}^{2}{u}_{{\rho }_{i}}^{2}+{c}_{{\rho }_{w}}^{2}{u}_{{\rho }_{w}}^{2}$$

where c_parameter_ are constant for a given sea ice configuration (FB, SD and densities).

Table 5 provides two floe examples, one FYI and one MYI, with respectively an ice freeboard of 0.10 m and 0.20 m, and a snow depth of 0.15 m and 0.35 m. The densities of the ice, the snow and the water and the associated uncertainties are taken respectively from^12,53,57^. As an example, for an uncertainty of 0.03 m on the measured FB we get SIT uncertainties of 0.82 m for the FYI floe and of 0.62 m for the MYI floe.

**Table 5:** Estimated SIT with its uncertainty (last line) for two typical sea ice configurations, FYI on left, MYI on right.

	FYI (FB_ice_ = 0.10 m)				MYI (FB_ice_ = 0.20 m)			
	mean	u	c^2^	c^2^u^2^	mean	u	c^2^	c^2^u^2^
**FB**_**laser**_**(m)**	0.25	0.03	91.6	0.08	0.55	0.03	52.0	0.05
**SD (m)**	0.15	0.09	47.1	0.38	0.35	0.09	26.7	0.22
**ρ**_**i**_ **(kg/m**^**3**^**)**	917	36.0	16.2 10^−5^	0.21	882	23.0	23.1 10^−5^	0.12
**ρ**_**s**_ **(kg/m**^**3**^**)**	290	3.2	19.7 10^−7^	0.00	290	3.2	60.8 10^−7^	0.00
**ρ**_**w**_ **(kg/m**^**3**^**)**	1024	0.5	13.9 10^−5^	0.00	1024	0.5	19.0 10^−5^	0.00
**SIT**	**1.36**	**0.82**			**2.16**	**0.62**		

**Fig. 11:**
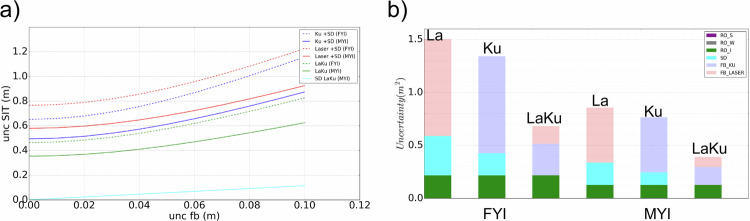
Left: Sea ice thickness uncertainties for a FYI (thin lines) and a MYI case (thick lines) as a function of the measured freeboard uncertainty using laser altimeter (in red), Ku altimeter (in dark blue), or combining Ku and Laser measurements (in green). For this last case we can also estimate the measured snow depth uncertainty (in light blue, the FYI and MYI curves are very close to each other, almost overlapping). The right part of this figure shows the relative contributions of each square uncertainty (u_param_ c_param_)^2^ on the SIT square uncertainty u^2^_SIT_ (cf Eq. 9) with FB laser and Ku contributions in pink and light purple, SD in light blue, ice density in green, snow density in dark purple, water density in grey (too small to be visible). The parameters used in this example are those given in Table 5.

We can observe from this table that the uncertainties coming from the density of snow and water have negligible effects. The main impacts come from SD, the ice density and the laser freeboard. Even if the measured FB was perfect (uncertainty = 0), because of the uncertainties on SD and on the ice density, the uncertainty on the SIT would remain of 0.77 m and 0.58 m for respectively the FYI and the MYI examples. More generally, the SIT uncertainty can be computed as a function of the measured FB uncertainty. The result is displayed Fig. 11 in red for the laser case.

The same methodology can be applied in the case of FB_Ku_ measurements with an auxiliary snow depth. We thus obtain the Eq. 11 below derived from Equation 5 and the resulting uncertainties are displayed in dark blue in Fig. 11.

11 $$\begin{array}{c}{u}_{{SIT}}^{2}={u}_{{{FB}}_{{Ku}}}^{2}{\left[\frac{{\rho }_{w}}{{\rho }_{w}-{\rho }_{i}}\right]}^{2}+{u}_{{SD}}^{2}{\left[\frac{{\rho }_{w}{\left(1+T{\rho }_{s}\right)}^{1.5}-{\rho }_{w}+{\rho }_{s}}{{\rho }_{w}-{\rho }_{i}}\right]}^{2}\\ +{u}_{{\rho }_{s}}^{2}{\left[\frac{1+1.5T{\rho }_{w}{\left(1+T{\rho }_{s}\right)}^{0.5}}{{\rho }_{w}-{\rho }_{i}}{SD}\right]}^{2}+{u}_{{\rho }_{w}}^{2}{\left[-\frac{{\rho }_{i}{{FB}}_{{Ku}}+{SD}\left({\rho }_{s}-{\rho }_{i}+{\rho }_{i}{\left(1+T{\rho }_{s}\right)}^{1.5}\right)}{{\left({\rho }_{w}-{\rho }_{i}\right)}^{2}}\right]}^{2}\\ +{\,u}_{{\rho }_{i}}^{2}{\left[\frac{{\rho }_{w}{{FB}}_{{Ku}}+{SD}\left({\rho }_{s}-{\rho }_{w}+{\rho }_{w}{\left(1+T{\rho }_{s}\right)}^{1.5}\right)}{{\left({\rho }_{w}-{\rho }_{i}\right)}^{2}}\right]}^{2}\end{array}$$

Finally, we applied the same method when FB_la_ and FB_Ku_ are both measured, and thus without need for auxiliary snow depth as it can be directly evaluated. Then instead of using an auxiliary SD product, we can compute both the SIT and SD and estimate the uncertainties for these two parameters. The corresponding uncertainty Eqs. 12 and 13, derived from Equation 7 and 6 respectively, are provided below.

12 $$\begin{array}{c}{u}_{{SIT}}^{2}={u}_{{{FB}}_{{Ku}}}^{2}{\left[\frac{-{\rho }_{w}-\left({\rho }_{s}-{\rho }_{w}\right)/(c/{c}_{s})}{{\rho }_{w}-{\rho }_{i}}\right]}^{2}+{u}_{{{FB}}_{{La}}}^{2}{\left[\frac{{\rho }_{w}+\left({\rho }_{s}-{\rho }_{w}\right)/(c/{c}_{s})}{{\rho }_{w}-{\rho }_{i}}\right]}^{2}\\ \,+{\,u}_{{\rho }_{s}}^{2}{\left[\frac{\left({{FB}}_{{La}}-{{FB}}_{{Ku}}\right)}{{\rho }_{w}-{\rho }_{i}}\left(1-\left({\rho }_{s}-{\rho }_{w}\right)1.5T{\left(1+T{\rho }_{s}\right)}^{-1}\right)\right]}^{2}\\ \,+{\,u}_{{\rho }_{w}}^{2}{\left[\frac{-{\rho }_{i}{{FB}}_{{La}}+\left({\rho }_{i}-{\rho }_{s}\right)\left({{FB}}_{{La}}-{{FB}}_{{Ku}}\right)/(c/{c}_{s})}{{\left({\rho }_{w}-{\rho }_{i}\right)}^{2}}\right]}^{2}\\ \,+{\,u}_{{\rho }_{i}}^{2}{\left[\frac{{\rho }_{w}{{FB}}_{{La}}+\left({\rho }_{s}-{\rho }_{w}\right)\left({{FB}}_{{La}}-{{FB}}_{{Ku}}\right)/{\left(1+T{\rho }_{s}\right)}^{1.5}}{{\left({\rho }_{w}-{\rho }_{i}\right)}^{2}}\right]}^{2}\end{array}$$

13 $${u}_{{SD}}^{2}={u}_{{{FB}}_{{Ku}}}^{2}{\left[\frac{{c}_{s}}{c}\right]}^{2}+{u}_{{{FB}}_{{La}}}^{2}{\left[\frac{{c}_{s}}{c}\right]}^{2}+{u}_{{\rho }_{s}}^{2}{\left[-\left({{FB}}_{{La}}-{{FB}}_{{Ku}}\right)1.5T{\left(1+T{\rho }_{s}\right)}^{-2.5}\right]}^{2}$$

The resulting uncertainties for the FYI and the MYI examples are displayed in Fig. 11 in green for the SIT uncertainty and in light blue for the SD uncertainty.

Figure 11 leads to three important observations:

Firstly, the uncertainties on the FYI (thin lines) are systematically higher than on the MYI (thick lines). This is essentially due to the fact that the density of the MYI is lower than that of the FYI, with 2 consequences: the first is that for the same ice thickness, the proportion of the ice that is above the surface, i.e. the freeboard, will be greater for MYI than for FYI, reducing the uncertainty of the ice draft and thus of the total thickness. Furthermore, the densities of ice and snow are closer together for MYI, so an error on the single values will affect less their ratio and thus have a smaller impact on the total ice freeboard which in turn will have less impact on the total uncertainty.

Secondly, we can also see that for the same uncertainty in the measurement of the freeboard, the FB_la_ has a larger impact than the FB_ku_ on the SIT uncertainty. This is due to the fact that Ku measures a freeboard that is closer to the actual ice freeboard, whereas the FB_la_ is the sum of the ice freeboard and the snow depth. However, this observation must be tempered by the fact that the FB_laser_ uncertainty estimated between 0.02 m and 0.04 m in^58^, is likely to be significantly smaller than the FB_ku_ uncertainty because of the smaller laser footprint and contrary to the Ku it is not impacted by the uncertainties on the penetration into the snow^48,59^ (not estimated here). In practice, this means that we have to consider a smaller uncertainty for the FB_laser_ than for FB_ku_, which leads to similar uncertainty on the SIT.

The third and final observation is probably the most important: the combination of laser and Ku measurements can provide a direct estimate of the SD with a very low uncertainty, which in turn leads to a very significant reduction (almost a factor of 2) in the total sea ice thickness uncertainty

^60^. Shows the potential of having dual-frequency colocated tracks. They study the CRYO2ICE (coincident IS2/CS2 tracks) snow depth variations across the winters 2020–2022 and the associated uncertainties. Intercomparisons have been performed against AMSR2, W99m and SnowModel-LG but unfortunately there are no *in situ* or airborne dataset which could be used as a reference. Further investigation based on new airborne and *in situ* data will be of critical importance for the CRISTAL mission which will be launched in 2028. This mission will carry a Ka/Ku dual-frequency altimeter to measure simultaneously the sea ice freeboard and the snow depth, which should lead to the most accurate estimates of sea ice thickness from radar altimetry to date.

## Data Availability

No custom code has been used in this study.
