# Correction: A multi-frequency altimetry snow depth product over Arctic sea ice

**DOI:** 10.1038/s41597-025-05293-1

**Published:** 2025-07-02

**Authors:** Alice Carret, Sara Fleury, Alessandro Di Bella, Jack Landy, Isobel Lawrence, Nathan Kurtz, Antoine Laforge, Jérome Bouffard, Tommaso Parrinello

**Affiliations:** 1 Serco, Frascati, Italy; 2 LEGOS, Université de Toulouse, IRD, CNES, CNRS, UPS, Toulouse, France; 3 ESA-ESRIN, Frascati, Rome, Italy; 4 UiT, Tromsø, Norway; 5 NASA Goddard Space Flight Center, Greenbelt, MD USA

**Keywords:** Physical oceanography, Cryospheric science

Correction to: *Scientific Data* 10.1038/s41597-024-04343-4, published online 17 January 2025

The authors would like to correct the following points:

**1) Corrections for a better understanding of the text**

Parameters in several equations, and the time periods referred to in some tables, were not clearly defined. These elements have been added to the text alongside explanations of how certain equations were derived.

**2) Corrections of figures in the text**

Incorrect ice freeboards sizes of 0.01m and 0.02m were stated on page 15, whereas the correct values are 0.10 and 0.20 m. These have been updated in the text.

**3) Corrections of ICEBird description**

The original ICEBird description provided incorrect numbers about the nominal fly and space between measurements as well as an incorrect link/reference to the data source. Figure 1 also depicted incorrect connectivity and locations of ICEBird tracks and has been updated.

**4) Corrections to other references**

The reference for the NESOSIM and ATL10 products were not the most recent. These have been updated.

**5) Addition of explanations for a better understanding**

Figures 7 and 8 suggest the alimentary dataset contains unphysical (negative) values, which is not explained in the original text. This paragraph was added to the technical validation section to explain this for a better understanding for readers:

Some negative values can be seen in Figure 7. This is mainly due to the fact that the orbits of the two satellites are different and, as a result, they do not acquire at the same date data from a given location. When the two freeboards are close in time, noise effects on the measurements can also lead to negative differences. In order to be able to carry out static analyses, we have chosen to keep all the measurements, but if this product is to be used, for example, to calculate the thickness of the ice from freeboard measurements, we recommend setting the negative values to zero.

An explanation of the assumption of the parameter’s independence in the uncertainty calculation (equation 9) was also added to support our demonstration and the following text updated on page 14 from:

If we consider that the different involved parameters are independent and their distributions are Gaussian, then the sea ice thickness uncertainties can be computed from partial derivation of Equ. 3:

To:

As a first step in this analysis, let's consider the case where snow depth is an auxiliary product derived from independent observations (eg, Warren 99, AMSR), a model (eg, NESOSIM, SnowModelLG, etc.), or any other source. With the additional assumption of a Gaussian distribution the sea ice thickness uncertainties can be computed from partial derivation of Equ. 3:

The same ambiguity was also present on page 16 and so an update was made to make the hypothesis more explicit.

**6) Corrections in Equations 9 and 12**

Equation 9: removed the 3 dots on the first line which could have raised questions

Equation 12: some missing parentheses: /c/cs replaced by /(c/cs) in 3 terms

The original article has been corrected.

